# Supplementary figures and images for: Do protection gradients explain patterns in herbivore densities? An example with ungulates in Zambia’s Luangwa Valley
Source: PLoS One. 2019 Oct 30;14(10):e0224438. doi: 10.1371/journal.pone.0224438 (PMC6821096; doi:10.1371/journal.pone.0224438)

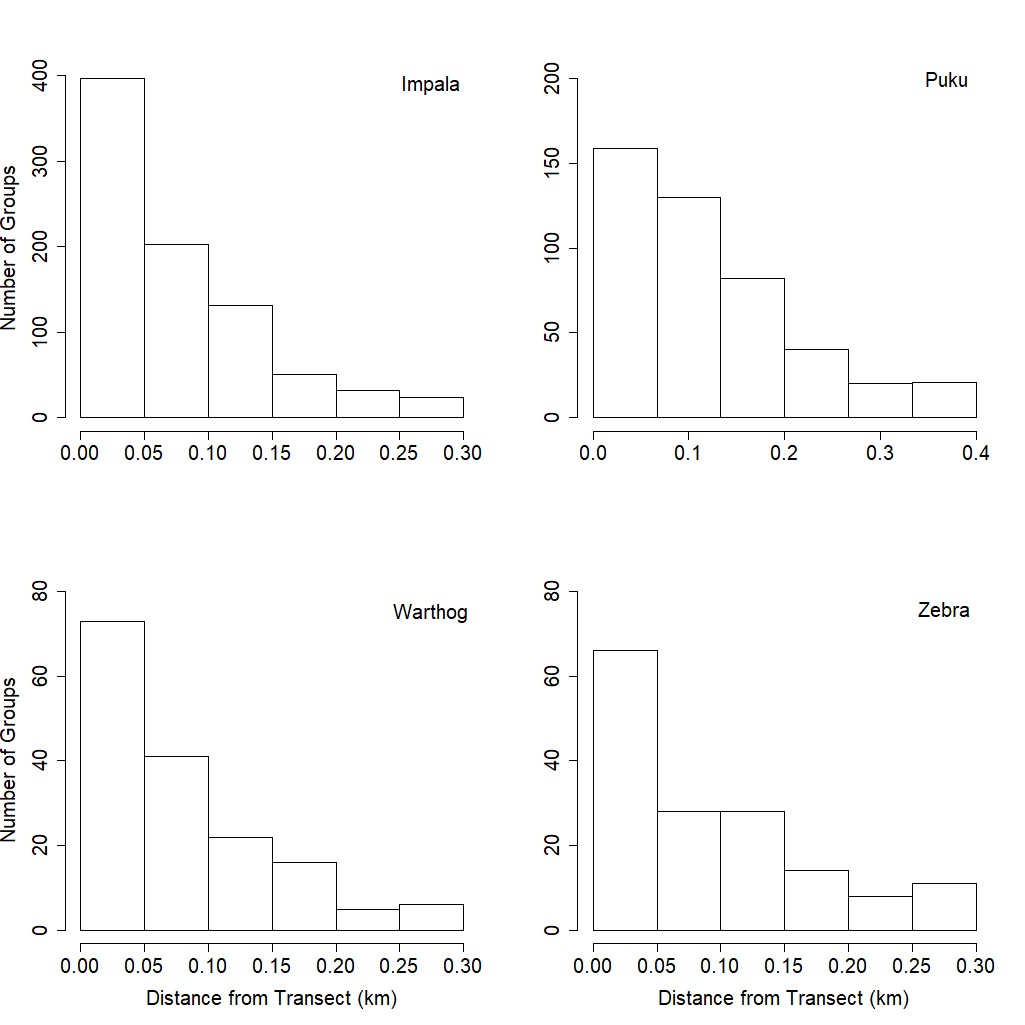

Supplement: S1 Fig — Distributions of detections of the four study ungulate species from driven line transects. Detection data are truncated to exclude outlier detections (400m for puku, 300m for impala, warthog, and zebra). (TIF) [file pone.0224438.s002.tif]
