# Supplementary material for: Do protection gradients explain patterns in herbivore densities? An example with ungulates in Zambia’s Luangwa Valley
Source: PLoS One. 2019 Oct 30;14(10):e0224438. doi: 10.1371/journal.pone.0224438 (PMC6821096; doi:10.1371/journal.pone.0224438)

PLOS ONE

Do protection gradients explain patterns in herbivore densities? An example with ungulates in Zambia’s Luangwa Valley

--Manuscript Draft--

**Manuscript Number:**	PONE-D-19-19082
**Article Type:**	Research Article
**Full Title:**	Do protection gradients explain patterns in herbivore densities? An example with ungulates in Zambia’s Luangwa Valley
**Short Title:**	Herbivore densities across a Zambian protection gradient
**Corresponding Author:**	Elias Rosenblatt<br>University of Vermont<br>Burlington, VT UNITED STATES
**Keywords:**	wildlife depletion; protected areas; Zambia; ungulates; human encroachment; protection gradient
**Abstract:**	Ungulate populations face declines across the globe, and populations are commonly conserved by using protected areas. However, assessing the effectiveness of protected areas in conserving ungulate populations has remained difficult. Using herd size data from four years of line transect surveys and distance sampling models, we modeled population densities of four important herbivore species across a gradient of protection on the edge of Zambia’s South Luangwa National Park (SLNP) while accounting for the role of various ecological and anthropogenic variables. Our goal was to test whether protection was responsible for density dynamics in this protection gradient, and whether a hunting moratorium impacted herbivore densities during the studies. For all four species, we estimated lower densities in partially protected buffer areas adjacent to SLNP (ranging from 4.5-fold to 13.2-fold lower) compared to protected parklands. Density trends through the study period were species-specific, with some species increasing, decreasing, or remaining stable in all or some regions of the protection gradient. Surprisingly, when controlling for other covariates, we found that these observed differences were not always detectably related to the level of protection or year. Our findings highlight the importance of accounting for variables beyond strata of interest in evaluating the effectiveness of a protected area. This study highlights the importance of comprehensively modeling ungulate population density across protection gradients, identifies lands within an important protection gradient for targeted conservation and monitoring, documents prey depletion and expands our understanding on the drivers in a critical buffer area in Zambia.
**Order of Authors:**	Elias Rosenblatt
	Scott Creel
	Paul Schuette
	Matthew Becker
	Dave Christianson
	Egil Dröge
	Thandiwe Mweetwa
	Henry Mwape
	Johnathan Merkle
	Jassiel M'soka
	Jones Masonde
	Twakundine Simpamba
**Additional Information:**	
**Question**	**Response**

Powered by Editorial Manager® and ProduXion Manager® from Aries Systems Corporation

**Financial Disclosure**<br><br>Enter a financial disclosure statement that describes the sources of funding for the work included in this submission. Review the submission guidelines for detailed requirements. View published research articles from *PLOS ONE* for specific examples.<br><br>This statement is required for submission and **will appear in the published article** if the submission is accepted. Please make sure it is accurate.<br><br>**Unfunded studies**<br>Enter: *The author(s) received no specific funding for this work.*<br><br>**Funded studies**<br>Enter a statement with the following details:<br>• Initials of the authors who received each award<br>• Grant numbers awarded to each author<br>• The full name of each funder<br>• URL of each funder website<br>• Did the sponsors or funders play any role in the study design, data collection and analysis, decision to publish, or preparation of the manuscript?<br>• **NO** - Include this sentence at the end of your statement: *The funders had no role in study design, data collection and analysis, decision to publish, or preparation of the manuscript.*<br>• **YES** - Specify the role(s) played.<br><br>* typeset	This research was funded by: 1. WorldWide Fund for Nature–Netherlands, https://www.wnf.nl/, Grantees: Matt Becker; 2. National Science Foundation Animal Behavior Program under IOS-1145749, Website: https://www.nsf.gov/funding/aboutfunding.jsp, Grantees: Scott Creel, Matt Becker, Dave Christianson, Paul Schuette; 3. National Geographic's Big Cats Initiative, Website: https://www.nationalgeographic.org/projects/big-cats-initiative/, Grantees: Matt Becker; 4. Painted Dog Conservation Inc, Website: http://www.painteddogconservation.iinet.net.au/, Grantees: Matt Becker. The funders had no role in study design, data collection and analysis, decision to publish, or preparation of the manuscript.
**Competing Interests**<br><br>Use the instructions below to enter a competing interest statement for this submission. On behalf of all authors, disclose any competing interests that could be perceived to bias this work—acknowledging all financial support and any other relevant financial or non-financial competing interests.<br><br>This statement **will appear in the published article** if the submission is accepted. Please make sure it is	The authors have declared that no competing interests exist.

accurate. View published research articles from *PLOS ONE* for specific examples.<br><br>**NO authors have competing interests**<br><br>Enter: *The authors have declared that no competing interests exist.*<br><br>**Authors with competing interests**<br><br>Enter competing interest details beginning with this statement:<br><br>*I have read the journal's policy and the authors of this manuscript have the following competing interests: [insert competing interests here]*<br><br>* typeset	
**Ethics Statement**<br><br>Enter an ethics statement for this submission. This statement is required if the study involved:<br><br>• Human participants<br>• Human specimens or tissue<br>• Vertebrate animals or cephalopods<br>• Vertebrate embryos or tissues<br>• Field research<br><br>Write "N/A" if the submission does not require an ethics statement.<br><br>General guidance is provided below. Consult the submission guidelines for detailed instructions. **Make sure that all information entered here is included in the Methods section of the manuscript.**	Though the animals involved in this submitted manuscript were only observed, covariate data were derived from radio-collared lionesses (Panthera leo) as part of a coinciding research project. This coinciding project was in accordance with animal welfare protocols set by the Zambia Department of Veterinary and Livestock Development, the country's regulatory authority.

**Format for specific study types**<br><br>**Human Subject Research (involving human participants and/or tissue)**<br>• Give the name of the institutional review board or ethics committee that approved the study<br>• Include the approval number and/or a statement indicating approval of this research<br>• Indicate the form of consent obtained (written/oral) or the reason that consent was not obtained (e.g. the data were analyzed anonymously)<br><br>**Animal Research (involving vertebrate animals, embryos or tissues)**<br>• Provide the name of the Institutional Animal Care and Use Committee (IACUC) or other relevant ethics board that reviewed the study protocol, and indicate whether they approved this research or granted a formal waiver of ethical approval<br>• Include an approval number if one was obtained<br>• If the study involved *non-human primates*, add *additional details* about animal welfare and steps taken to ameliorate suffering<br>• If anesthesia, euthanasia, or any kind of animal sacrifice is part of the study, include briefly which substances and/or methods were applied<br><br>**Field Research**<br><br>Include the following details if this study involves the collection of plant, animal, or other materials from a natural setting:<br>• Field permit number<br>• Name of the institution or relevant body that granted permission	
**Data Availability**<br><br>Authors are required to make all data underlying the findings described fully available, without restriction, and from the time of publication. PLOS allows rare exceptions to address legal and ethical concerns. See the PLOS Data Policy and FAQ for detailed information.	Yes - all data are fully available without restriction

Powered by Editorial Manager® and ProduXion Manager® from Aries Systems Corporation

A Data Availability Statement describing where the data can be found is required at submission. Your answers to this question constitute the Data Availability Statement and **will be published in the article**, if accepted.<br><br>**Important:** Stating 'data available on request from the author' is not sufficient. If your data are only available upon request, select 'No' for the first question and explain your exceptional situation in the text box.<br><br>Do the authors confirm that all data underlying the findings described in their manuscript are fully available without restriction?	
**Describe where the data may be found in full sentences. If you are copying our sample text, replace any instances of XXX with the appropriate details.**<br><br>• If the data are **held or will be held in a public repository**, include URLs, accession numbers or DOIs. If this information will only be available after acceptance, indicate this by ticking the box below. For example: *All XXX files are available from the XXX database (accession number(s) XXX, XXX.).*<br>• If the data are all contained **within the manuscript and/or Supporting Information files**, enter the following: *All relevant data are within the manuscript and its Supporting Information files.*<br>• If neither of these applies but you are able to provide **details of access elsewhere**, with or without limitations, please do so. For example:<br><br>*Data cannot be shared publicly because of [XXX]. Data are available from the XXX Institutional Data Access / Ethics Committee (contact via XXX) for researchers who meet the criteria for access to confidential data.*<br><br>*The data underlying the results presented in the study are available from (include the name of the third party*	Distance sampling data used in this analysis are available from the Dryad Digital Repository (accession number to be determined).

and contact information or URL).<br>• This text is appropriate if the data are owned by a third party and authors do not have permission to share the data.<br><br>* typeset	
Additional data availability information:	Tick here if the URLs/accession numbers/DOIs will be available only after acceptance of the manuscript for publication so that we can ensure their inclusion before publication.

Powered by Editorial Manager® and ProduXion Manager® from Aries Systems Corporation

Manuscript

Click here to download Manuscript Herbivore densities across protection gradients_Manuscript.docx

Do protection gradients explain patterns in herbivore densities? An example with ungulates in Zambia's Luangwa Valley.

Elias Rosenblatt[1,2,#a,&,*], Scott Creel[1,2, ¶,&], Paul Schuette[1,3, ¶,&], Matthew S. Becker[1,2, ¶,&], David Christianson[1,4, ¶,&], Egil Dröge[5,&,ʎ], Thandiwe Mweetwa[1,ʎ], Henry Mwape[1,ʎ], Johnathan Merkle[1,ʎ], Jassiel M'soka[1,ʎ], Jones Masonde[6,ʎ], Twakundine Simpamba[6,ʎ]

[1]Zambian Carnivore Programme, Mfuwe, Eastern Province, Zambia

[2]Department of Ecology, Montana State University, Bozeman, Montana, United States of America

[3]Alaska Center for Conservation Science, University of Alaska Anchorage, Anchorage, Alaska, United States of America

[4]School of Natural Resources and the Environment, University of Arizona, Tucson, Arizona, United States of America

[5]Wildlife Conservation Research Unit, University of Oxford, Tubney, United Kingdom

[6]Department of National Parks and Wildlife, Chilanga, Zambia

[#a]Current Address: Rubenstein School of Environment and Natural Resources, University of Vermont, Burlington, Vermont, United States of America

*Corresponding Author

E-mail: erosenbl@uvm.edu (ER)

¶ Contributed to the Funding Acquisition, Methodology, and Project Administration, and Supervision

& Contributed to the data curation, formal analysis, and original draft preparation and editing

ʎ Contributed to the methodology and writing through review, and editing

Abstract

Ungulate populations face declines across the globe, and populations are commonly conserved by using protected areas. However, assessing the effectiveness of protected areas in conserving ungulate populations has remained difficult. Using herd size data from four years of line transect surveys and distance sampling models, we modeled population densities of four important herbivore species across a gradient of protection on the edge of Zambia's South Luangwa National Park (SLNP) while accounting for the role of various ecological and anthropogenic variables. Our goal was to test whether protection was responsible for density dynamics in this protection gradient, and whether a hunting moratorium impacted herbivore densities during the studies. For all four species, we estimated lower densities in partially protected buffer areas adjacent to SLNP (ranging from 4.5-fold to 13.2-fold lower) compared to protected parklands. Density trends through the study period were species-specific, with some species increasing, decreasing, or remaining stable in all or some regions of the protection gradient. Surprisingly, when controlling for other covariates, we found that these observed differences were not always detectably related to the level of protection or year. Our findings highlight the importance of accounting for variables beyond strata of interest in evaluating the effectiveness of a protected area. This study highlights the importance of comprehensively modeling ungulate population density across protection gradients, identifies lands within an important protection gradient for targeted conservation and monitoring, documents prey depletion and expands our understanding on the drivers in a critical buffer area in Zambia.

1. Introduction

In sub-Saharan Africa, ungulates have ecological and economic value through their top-down effects on plant communities and their bottom-up effects as prey for large carnivores [1]. Despite their importance, many protected ungulate populations have recently declined at a rate comparable to populations with less protection [2,3] and face rapid human encroachment [4]. Protected areas (PAs) are an important tool to protect wildlife from human activities and reduce human-wildlife conflict [5]. Strictly protected areas

with no permanent settlements and no consumptive use are often bordered by areas with some lower level of protection, creating a gradient to buffer edge effects [6] and source-sink dynamics [7]. However, it remains unclear how effective these protection gradients are in protecting ungulate species of ecological importance or conservation concern [5].

Despite the intuitive benefits of PAs, assessing the effectiveness of PAs in protecting ungulate populations is difficult. First, to provide a valid test of the effect of protection gradients, studies must control for ecological differences between PAs and adjacent buffer zones that could affect ungulate density and distribution. Protected areas are not randomly distributed [8] but instead are generally placed where wildlife densities are high, while buffer zones are often designated in areas with lower wildlife densities. Thus, differences in animal density between PAs and buffer zones can exist due to natural ecological differences between locations unrelated to the effectiveness of their protection. Second, ungulates cannot be surveyed with perfect detection in most habitats [9]. Methods exist to account for imperfect detection when estimating animal densities [10,11], but many studies of protection gradients assume perfect detection or use an index to convert counts that rely on untested assumptions about detection probability. Finally, even when methods that account for detection are used, a common approach is to model the density of groups with distance sampling [10] and then convert group density to individual density using a mean group size [10, but see 12]. This conversion uses either mean group size across all observations or across focal categorical variables, such as vegetation types [13]. Because ungulate group size is typically influenced by the same variables that affect the distribution of herds, this approach may not be entirely accurate. Important broad studies have aided our understanding of the effectiveness of PAs to conserve ungulates [14-17], but their inferences have been constrained by one or more of these limitations.

Zambia contains several PAs important for regional conservation of ungulates and the large carnivores that depend on ungulate prey [18], and most of these PAs face human encroachment that is approaching rapidly or already has reached the PA itself [19]. Most Zambian PAs are buffered by Game Management

Areas (GMAs) that allow some human settlement and support consumptive uses of wildlife and resources, such as legal trophy hunting [20]. Rapid human population growth in GMAs has brought increased pressures of illegal bushmeat harvest and habitat conversion, challenging the effectiveness of Zambia's GMAs as buffers for adjacent PAs [21]. South Luangwa National Park (SLNP) is Zambia's premier PA for wildlife-based photo-tourism and conserves regionally-important populations of several threatened and endangered species yet faces rapid human pressure from adjacent GMAs [19]. Recent studies of large carnivore demography and dynamics [20,22-25] and studies of bushmeat poaching patterns [21,26,27] suggest that the depletion of ungulate populations from bushmeat poaching may be affecting the ecological integrity of this protection gradient. Aerial surveys also suggest overall ungulate declines in the Luangwa Valley and lower ungulate density in GMAs compared to PAs [21], but these studies are generally inconclusive due to low precision of density estimates and no correction for variability in detection. Finally, it has been suggested that a temporary moratorium in all trophy hunting from 2013 to 2014 allowed increased poaching activity in the absence of the primary wildlife-based tourism activity in GMAs [28]. Despite this concern and reported increased poaching in communities in GMAs adjacent to SLNP [29], there has not been sufficient data to test the relationship between the hunting moratorium and herbivore population trends. Therefore, to better inform management actions in this important PA, there is a clear need for unbiased and precise estimate of ungulate abundance in SLNP and adjacent GMAs, for tests of the relationship between ungulate density and the level of protection, and for tests of trends in density over time and across management actions, while addressing the difficulties introduced above.

Here we use data from repeated, stratified line transect sampling to fit distance sampling and group size models to estimate population densities of impala (*Aepyceros melampus*), puku (*Kobus vardonii*), plains zebra (*Equus quagga*), and warthog (*Phacochoerus africanus*) across the South Luangwa Protection Gradient from 2012-2015. These species are abundant in the area, important for ecosystem health, primary targets for illegal bushmeat trade and are expected to be negatively impacted by the absence of

trophy hunting during the 2013-2014 moratorium. For each species we model both group density and group size as functions of top-down, bottom-up, abiotic, and anthropogenic covariates, and then estimate differences in population density across space and time while controlling for these effects. Our approach addresses the above difficulties in assessing the efficacy of protected areas as it accounts for imperfect detection, robustly integrates ecological and anthropogenic covariates that impact both the distribution and size of ungulate groups, compares ungulate densities within a PA and GMA that are similar ecologically but differ in human usage, and assess population trends with the temporary cessation of trophy hunting activities. The results improve our understanding of the efficacy of protection gradients in buffering the impacts of human encroachment and bushmeat poaching and provide baseline estimates of density for ecologically important ungulate populations that face increasing anthropogenic pressures.

2. Materials and Methods

2.1 Study Area

Our study area (hereafter the South Luangwa Protection Gradient, or SLPG) was within a 1 200 km^2 complex of grasslands, scrublands, and forests situated along the perennial Luangwa River, the primary eastern boundary of SLNP and the adjacent Lupande GMA (hereafter the GMA; Figure 1) [30,31]. The Luangwa River is only a seasonal barrier for wildlife and attracts the highest densities of wildlife in the area as it is the largest and most reliable perennial water source in the region, particularly during the dry season (May-November).

Fig 1: Our intensive study area (1 200 km^2) on the eastern boundary of South Luangwa National Park (SLNP; S13.07958 E31.77407), Zambia, faceted by key spatial covariates. Line transects (n=15) were stratified across bottom-up, top-down, anthropogenic, and abiotic covariates, and each transect was comprised of multiple segments (n=97; not shown). Maps are faceted to illustrate key spatial covariates. Group observations and covariates were recorded by segment (see Section 2.2.2)

The SLPG includes three regions of interest for this study. West of the Luangwa River are fully protected SLNP lands, where the primary human activity is photographic safari tourism and Park management activities. East of the Luangwa River there is a section of SLNP that is also popular for photographic tourism but is thought to be exposed to more illegal bushmeat poaching due to open borders and a public road that bisects the area. While this is a small, unsurfaced dirt track, it supports considerable local foot and bicycle traffic, and some vehicular traffic. Finally, the GMA lands, also east of the Luangwa River, contain a growing human population (annual growth of 3.8%) [32] that has raised an array of conservation concerns (see Section 1). Trophy hunting operates in leased concessions in the GMA (save for a moratorium from 2013-2014) [20], targeting an array of wildlife species including those studied here [30]. In short, our study area encompasses areas of high conservation importance across a protection gradient with associated variation in human influence and other potentially limiting factors.

2.2 Study Design

2.2.1 Survey Design

We used line transects and distance sampling [10] to estimate herd density (herds/km^2) and herd size (individuals/herd) for ungulates across the SLPG. We surveyed 15 transects by vehicle across our study area (120.5 km in total), with sufficient spacing between transects to minimize the risk of double-counting groups [10] (Figure 1). We established most transects in a generally east-west orientation (i.e., perpendicular to the Luangwa River) to sample across the range of environmental and anthropogenic variables of interest. Topography and vegetation impassible by vehicle forced us to follow roads for sections of transects, which we accounted for in our modeling of detection (See Section 2.2.2). Each transect was split into segments defined by observed changes in vegetation cover type or a maximum length of 2km, resulting in a total of 97 segments across the three regions of interest in the SLPG (West of the Luangwa and National Park, n = 62; East of the Luangwa and National Park, n = 15; GMA, n = 20). These segments allowed us to estimate the role of covariates on a finer scale than for entire transects (e.g. distance to the Luangwa River; See Section 2.2.2). We were not able to achieve balanced sampling across

the SLPG due to human settlement and agriculture activity in the GMA. We recorded herbivore group observations and covariate values on the segment level as required by our analysis described below.

We conducted 10 surveys of all 97 segments during daylight hours (0900-1700) at the beginning (June), middle (August), and end (October) of the dry season (May-November) from 2012-2015 (n = 970 segment-surveys). June 2012 and August 2015 were not surveyed due to logistic constraints. We could not conduct any surveys during the rainy season (December-April) as much of the study area is inaccessible. During each survey, the vehicle was driven at a maximum speed of 15 km/h, with two observers seated on the rooftop scanning for animal herds. When a herd or single animal was seen, the observers recorded the species composition and size of the herd, and the distance (aided by a laser rangefinder) and azimuth to the herd following standard distance sampling protocol [10]. We reduced our risk of double-counting animal herds during each survey by surveying all segments over a period of 3-4 days.

2.2.2 Factors Affecting Group Density and Size

We considered a suite of bottom-up, top-down, anthropogenic, and abiotic variables that could affect herd density and herd size of the focal species on the segment level (Table 1). We measured ten covariates that characterize bottom-up effects of vegetation composition and structure on herd density and herd size. While surveying segments we recorded the presence or absence of green grass and of grassy lagoon patches, and the predominant grass height category (short=<10cm, intermediate: 10-100cm, long: >100cm). We suspected that vegetation composition would influence both herd density and herd size, and our ability to detect herds. To model detection, we assigned each segment to one of six vegetation structure categories, as well as one of three simplified vegetation categories (Table 1): because transects were split into segments at irregular intervals that were defined by changes in vegetation structure, each segment was relatively homogenous in structure. We considered these two structure covariates separately during our analyses as they were two descriptions of the same underlying variable. To model group density and herd size, we classified vegetation composition and heterogeneity within a 240m-wide buffer

around each segment (hereafter buffer) to characterize vegetation beyond the view of the survey team. Using Landsat 7 Global Land Survey 2010 imagery we partitioned pixels into 10 vegetation classes of differing vegetation densities using the clara() function in the *cluster* package [34] in R [35]. We then combined vegetation classes into four cover types based on vegetation density and validated these cover type classifications during our line transect surveys. These cover types included closed scrubland (right-skewed, and therefore log-transformed), closed woodland, open woodland, and open grassland. We estimated the proportion of buffer composed of each cover type for each segment using the *sp* package in R [36,37] and estimated the density of edges between cover types within each buffer (km edge/km^2) using the perimeter tool in QGIS 2.0.1 (www.qgis.org).

Table 1: A summary of all covariates that were thought to impact herbivore density, classified as Bottom-up (B), Top-down (T), Anthropogenic (A), or Abiotic (AB). Covariates were considered in distance sampling models to impact group super-population (λ), availability for sampling (φ), or detection (p). Covariates were (Y) or were not (N) considered in models predicting group size.

Covariate	Factor Class	Categorical Levels	Continuous Range (Mean)	Distance Sampling	Group Size
Edge Density (km/km2)	B	-	3.5 - 55.6 (24.4)	λ	Y
Grassy Lagoon	B	Present, Absent	-	φ	Y
Percent Closed Scrub	B	-	0 - 38.0 (2.3)	λ	Y
Percent Closed Woodland	B	-	0 - 90.8 (23.4)	λ	Y
Percent Open Woodland	B	-	0.5 - 96.3 (37.7)	λ	Y
Percent Open Grassland	B	-	0 - 99.5 (36.7)	λ	Y
Grass Height	B	Short, Intermediate, Tall	-	φ	Y
Grass Color	B	Green, Brown	-	φ	Y
Vegetation/Density Class	B	Closed Scrub, Closed Woodland, Open Scrub, Open Woodland, Open Grassland Flooded, Open Grassland Not Flooded	-	p	N
Simplified Vegetation Class	B	Scrub, Woodland, Grassland	-	P	N
Lion UD	T	-	6.0-121.1 (49.0)	λ	Y
Distance to Roads (km)	A	-	0-2.4 (0.3)	λ	Y
Side of River	A	East, West	-	λ	Y
Area	A	GMA, Park	-	λ	Y
Segment Path Type	A	Off-road, Seasonal Track, Gravel Road	-	p	N
Distance to Luangwa River (km)	AB	-	0.2-14.7 (4.1)	λ	Y
Distance to Seasonal Stream (km)	AB	-	0-9.4 (3.4)	λ	Y
Water	AB	Present, Absent	-	φ	Y
Burn	AB	Present, Absent	-	φ	Y
Year	AB	2012, 2013, 2014, 2015	-	φ	Y
Dry Season Stage	AB	Early, Middle, Late	-	φ	Y

Differences in predation risk could affect herd density or herd size across the SLPG, thus we quantified predation risk across our study area by measuring the utilization of the area by the lion (*Panthera leo*) population (the most abundant large carnivore within the study area) [20]. We fit a single kernel utilization distribution to 7 785 lion locations collected over five years (2008-2012) from lions equipped with GPS radiocollars and direct observation of 18 lion prides and 14 male coalitions [20]. Though this

spatial information overlaps only one year of this study, ongoing lion studies indicate little change in lion distribution [25]. We used *sp*, *rgdal* [38], and *plyr* [39] packages in R to estimate the distribution of daily distance moved from six lionesses equipped with GPS radiocollars (range: 0m - 17 300m) and used the 90th percentile (6 974m) as a smoothing parameter for the utilization distribution (UD) [40]. We fit the UD using the bivariate normal kernel function in the *adehabitatHR* package [41], with an output grid of 300m x 300m. We converted the UD to a raster using the *raster* [42] and *sp* packages and extracted and standardized lion UD values for each segment's midpoint, thereby quantifying the risk of herbivores encountering the dominant large carnivore species in each segment.

The impact of anthropogenic activities was characterized for each segment by classifying whether it lay within the PA or partially-protected GMA (hereafter protection), on the east or west side of the Luangwa River (hereafter side), and the distance from the segment midpoint to the nearest road (also right-skewed, and log-transformed). We could not test for an interaction between protection and side as there are no GMA areas within the study area on the western side of the Luangwa River. We also could not include distance to park boundary in our analysis as this boundary is mostly defined by the Luangwa River. Despite the Luangwa River defining a boundary in the SLPG, any effect of this boundary in our study is confounded by the abiotic influence of the Luangwa River as the SLPG's perennial water source. Therefore, we chose to treat the Luangwa River as an abiotic variable, and not an anthropogenic variable (see below). Finally, as some segments followed roads, we classified each segment's path type to account for any biases in detection for road-based segments (Table 1) [43].

We measured six abiotic covariates to characterize the environmental conditions potentially influencing herbivore density and distribution in the Luangwa Valley (Table 1). Availability of water is a limiting factor for wildlife in the Luangwa Valley, with water sources diminishing and disappearing with the progression of the dry season. Therefore, we measured distances from each segment's midpoint to the perennial Luangwa River and the nearest seasonal stream and recorded the dry season stage and whether standing water was present during surveys of each segment. We also recorded the year of each survey to

determine annual trends in group density and size. Finally, we recorded whether there was evidence of a fire that had burned through the segment for each survey, as post-fire "green flush" provides herbivores with high-quality forage and potentially reduces predation risk [44].

2.3 Analytical Methods

2.3.1 Herd Density Analysis

We used a multinomial generalized distance sampling model to estimate the 'super-population' of herds (λ) for each species within each segment, while accounting for imperfect detection (*p)* and varying availability for detection at the time of the survey (φ)[45]. We used the gdistsamp() function to fit candidate models for each species in the *unmarked* package [45] in R. We truncated herd sighting data for each species to exclude outlier distances that would compromise estimation of detection probability.

To focus on accurate estimation of the parameter of interest (group density), we evaluated a set of models in three steps using Akaike's Information Criteria (AIC; Table 2). We first identified the best-supported model for detection across all combinations of covariates thought to influence detection using hazard, half-normal, and uniform detection functions, while estimating only a mean for availability and super-population (Table 1 and 2). Using the best-supported detection model and a uniform super-population model, we next identified the best supported model for availability across all combinations of covariates thought to influence availability (Table 1 and 2). In the third step, while modeling detection and availability using their best-supported models, we used AIC model selection to identify the best supported models ($\leq$ 1 AIC) for each of the four types of effect on the super-population of herds: bottom-up, top-down, anthropogenic, and abiotic (Table 2). Finally, we took the top model for each of these four types and used AIC scores to identify the final density models ($\leq$ 1 AIC), selecting from a set with all additive combinations of effects in the top models for each type (Table 2). This multi-stage approach to limit the number of models compared was necessary to reduce computation time. We used deviance goodness-of-fit tests for each species' top herd density model(s) to examine model fit. We used these final models and

model averaging (when more than one model received comparable support from the data) to estimate herd super-population (λ) and availability (φ) using the *predict()* function, and derive herd density estimates ($\widehat{D}$) as the product of these two parameter estimates.

Table 2: Full models of detection (*p*), availability (*φ*), and group super-population (λ) to illustrate the model refinement process. We used AIC model selection to evaluate these models and their subsets for each species and used the best supported models for each parameter to build the final model set. For continuous covariates we considered linear, log, and second-order polynomial relationships. For super-population, we identified the best performing model for each covariate type, and then created a final candidate model list using all combinations of those parametrizations. We split bottom-up abundance model selection into vegetation availability (proportion of vegetation classifications around segments) and edge density model sets to reduce computation time.

Step	Parameter	Detection	Availability	Super-population
1	*p*	~ Vegetation/Density Class + Path Type **or** ~ Vegetation Class + Path Type	~1	~1
2	*φ*	*p(Top)*	~Dry Season Stage + Year + Grass Height + Grass Color + Burn + Grassy Lagoon + Water	~1
3	λ(Abiotic)	*p(Top)*	*φ(Top)*	~ Distance to Luangwa River + Distance to Seasonal Stream
	λ(Bottom-Up: Edge Density)	*p(Top)*	*φ(Top)*	~ Edge Density
	λ(Top-Down)	*p(Top)*	*φ(Top)*	~ Lion UD
	λ(Bottom-Up: Vegetation Availability)	*p(Top)*	*φ(Top)*	~ log(% Closed Scrub) + % Closed Woodland + % Open Woodland + % Open Grassland
	λ(Anthropogenic)	*p(Top)*	*φ(Top)*	~ log(Distance to Roads) + Area + Side of River
	**λ(Final)**	***p(Top)***	***φ(Top)***	**~ λ(Abiotic-*Top*) + λ(Edge Density-*Top*) + λ(Top-Down-*Top*) + λ(Vegetation Availability-*Top*) + λ(Anthropogenic-*Top*)**

2.3.2 Herd Size Analysis

We fit zero-truncated Poisson (ZTP) regression models using the *vglm()* function in the VGAM package [46,47] in R to estimate segment-specific mean herd sizes for the four focal species while controlling for the effects of covariates described above (Table 1). First, we used a ZTP model to test whether herd size was affected by distance from the transect, as is expected if herd size affects detection, and only used herd size observations at 0m from the transect if this there was evidence of this effect ($p < 0.15$) [10]. Next, we dropped covariates with badly imbalanced sampling or that were highly correlated with other covariates (Pearson's $r > 0.6$) from consideration. We then used reverse step-wise likelihood-ratio (LR) tests to select a herd size model for each species, and then confirmed this model selection using forward step-wise LR tests. We evaluated model fit by fitting a linear regression of Pearson's residuals on predicted herd sizes and assumed adequate fit if the estimated intercept and slope were not detectably different from 0 [46]. Using the final ZTP model and the *predict()* function we estimated differences in herd size across covariate ranges and variation in herd size while accounting for the non-random distribution and correlation of covariates across the study area. To avoid extrapolation, we only estimated mean herd size for segments with covariate values within the range documented during herd observations.

2.3.3 Population Density Analysis

To estimates population densities for each species, we multiplied mean herd density by estimates of mean herd size for each segment and used non-parametric bootstrapping to estimate mean population density and its variance across SLPG regions and years. We also predicted herd density and herd size with all covariates other than protection, side, and year fixed at their mean value, estimated mean population density and variance across those strata of interest, and calculated differences across SLPG regions and across years. In summary, our comprehensive approach allows a test for the effect of protection and year on population density that does not ignore the ecological and abiotic differences across the protection gradient.

3. Results

3.1 Herd Density and Herd Size

During the 10 surveys of 97 segments, we detected 890 impala herds, 478 puku herds, 175 zebra herds, and 169 warthog herds. After truncating datasets to maintain suitable detection probabilities (400m for puku, 300m for the other three species), our final sample sizes for our herd density analysis were 836 impala herds, 452 puku herds, 155 zebra herds, and 163 warthog herds. Distance had a positive association with impala and puku herd size ($p<0.0001$ and $p=0.005$, respectively), but not with zebra and warthog herd size ($p=0.43$ and $p=1$, respectively). These results indicated that large impala and puku herds were more detectable at large distances, so to avoid bias we only used herds that were directly on the transect to estimate herd size for these two species. Our final sample for our herd size analysis included 122 impala herds (mean=7.1 individuals, range: 1-75), 56 puku herds (mean=6.6 individuals, range: 1-111), 155 zebra herds (mean=5.1 individuals, range: 1-24), and 163 warthog herds (mean= 2.6 individuals, range: 1-9).

The best supported herd density model(s) for each parameter varied between species (Table 3), and coefficient estimates varied in magnitude and sign between models (Table 4). Despite this variation two variables of primary interest - side of Luangwa River and protection status - were included in top herd density models for all species. Herd density was higher in the National Park for all species. Segments west of the Luangwa River were estimated to have higher impala and zebra herd densities, whereas puku and warthog densities were lower or not detectibly different than in segments east of the Luangwa River, respectively. Survey year influenced availability for zebra and warthog herd density, with herd density detectably higher for zebra in 2015, but detectably lower for warthog in 2015.

Table 3: Model selection results for distance sampling models estimating group density for the focal herbivore species. Model-averaged predictions were made using models within 1 delta AIC score of the top model; top models were used if there were no closely-competing models. Continuous covariates may appear in models as a linear, logarithmic (log), or 2nd-order polynomial (poly) association with the parameter.

Model - λ	Model - φ	Model - p	Parameters	delta AIC	AIC Weight
**Impala**					
~poly(distance to seasonal stream)+ poly(Distance to Luangwa River) + poly(edge density)+log(lion UD)+log(distance to road)+area+side of river	~dry season stage + grass height + burn + lagoon + water	~Vegetation/ Density	26	0	0.72
~poly(distance to seasonal stream)+ poly(Distance to Luangwa River) + poly(edge density)+log(lion UD)+ log(% Open Grassland)+ log(distance to road)+area+side of river	~dry season stage + grass height + burn + lagoon + water	~Vegetation/ Density	27	1.86	0.28
**Puku**					
~poly(distance to seasonal stream)+ poly(Distance to Luangwa River) + poly(edge density)+poly(lion UD)+log(CW)+log(OG)+poly(log(distance to road))+area+side of river	~grass height + grass color + burn + lagoon	~Vegetation+ Path	27	0	0.44
~poly(distance to seasonal stream)+ poly(Distance to Luangwa River) + poly(edge density)+poly(lion UD)+log(CW)+log(OG)+log(CS)+ poly(log(distance to road))+area+side of river	~grass height + grass color + burn + lagoon	~Vegetation+ Path	28	1.07	0.26
~poly(distance to seasonal stream)+ poly(Distance to Luangwa River) + poly(lion UD)+log(CW)+log(OG)+poly(log(distance to road))+area+side of river	~grass height + grass color + burn + lagoon	~Vegetation+ Path	25	1.61	0.20
**Zebra**					
~log(Edge) + log(CW) + log(OW) + area + side of river	~dry season stage + year + grass color + lagoon	~Vegetation/ Density + Path	23	0	0.13
~log(CW) + log(OW) + area + side of river	~dry season stage + year + grass color + lagoon	~Vegetation/ Density + Path	22	0.37	0.10
~log(distance to road)+log(CW) + log(OW) + area + side of river	~dry season stage + year + grass color + lagoon	~Vegetation/ Density + Path	23	0.58	0.09
~log(Edge) + log(CW) + log(OW) + log(distance to road) + area + side of river	~dry season stage + year + grass color + lagoon	~Vegetation/ Density + Path	24	0.7	0.09
~log(Edge) + log(CW) + log(OW) + poly(log(distance to road)) + area + side of river	~dry season stage + year + grass color + lagoon	~Vegetation/ Density + Path	25	1.43	0.06
~log(CW) + log(OW) + poly(log(distance to road)) + area + side of river	~dry season stage + year + grass color + lagoon	~Vegetation/ Density + Path	24	1.56	0.06
**Warthog**					
~log(Lion_UD)+poly(log(CS))+poly(CW)+poly(OG)+log(distance to road)+Area	~year + water	~Vegetation/ Density	22	0	0.10
~log(Lion_UD)+poly(CW)+poly(OW)+poly(OG)+log(distance to road)+Area	~year + water	~Vegetation/ Density	22	0.74	0.07
~log(Lion_UD)+poly(CW)+poly(OW)+poly(OG)+Area	~year + water	~Vegetation/ Density	21	1.11	0.06
~log(Lion_UD)+poly(log(CS))+poly(CW)+poly(OG)+Area	~year + water	~Vegetation/ Density	21	1.14	0.06
~Distance to Seasonal Streams+log(Lion_UD)+ poly(log(CS))+poly(CW)+ poly(OG)+log(distance to road)+Area	~year + water	~Vegetation/ Density	23	1.76	0.04
~Lion_UD+poly(log(CS))+poly(CW)+poly(OG)+log(distance to road)+Area	~year + water	~Vegetation/ Density	22	1.85	0.04

Table 4: Coefficient estimates from each species' top group density model. Coefficient estimates with p-values<0.05 are indicated (*). Covariates included in model averaging for zebra and warthog but not in the top model are also indicated (+).

	Impala	Puku	Zebra	Warthog
λ - Covariates	β Estimate (SE)	β Estimate (SE)	β Estimate (SE)	β Estimate (SE)
Intercept	-3.53 (0.98)*	-0.43 (0.45)	0.43 (0.99)	-1.53 (1)
B-Edge Density	2.36 (0.55)*	1.71 (1.19)	-	-
B-Edge Density^2	0.70 (0.46)	0.82 (0.8)	-	-
B-Log(Edge Density)	-	-	-0.49 (0.31)	-
B-Log(% CS)	-	-	-	-0.46 (1.17)
B-Log(% CS)^2	-	-	-	-2.57 (1.02)*
B-% CW	-	-	-	1.31 (2.11)
B-% CW^2	-	-	-	-2.55 (1.25)*
B-log(% CW)	-	-0.08 (0.02)*	-0.07 (0.02)*	-
B-% OW	-	-	-	+
B-% OW^2	-	-	-	+
B-log(% OW)	-	-	0.07 (0.15)	-
B-% OG	-	-	-	0.82 (1.67)
B-% OG^2	-	-	-	3.92 (1.21)*
B-log(% OG)	-	-0.01 (0.06)	-	-
T-Lion UD	-	-0.62 (2.2)	-	-
T-Lion UD^2	-	-3.96 (1.29)*	-	-
T-log(Lion UD)	1.34 (0.25)*	-	-	0.46 (0.22)*
A-Log(Distance to Roads)	-0.06 (0.02)*	-2.92 (1.17)*	+	0.08 (0.04)
A-Log(Distance to Roads)^2	-	-7.73 (1.64)*	-	-
A-Side: West	0.60 (0.25)*	-1.67 (0.42)*	0.71 (0.37)	-
A-Area: Park	0.51 (0.28)	2.34 (0.48)*	1.94 (0.69)*	1.56 (0.47)*
AB-Distance to Luangwa	-1.68 (1.20)*	-11.96 (3.09)*	-	-
AB-Distance to Luangwa^2	3.90 (0.78)*	8.22 (1.8)*	-	-
AB-Distance to Seasonal Stream	-0.01 (0.73)	1.04 (1.34)	-	-
AB-Distance to Seasonal Stream^2	-0.23 (0.61)	2.29 (1.03)*	-	-
φ - Covariates	β Estimate (SE)	β Estimate (SE)	β Estimate (SE)	β Estimate (SE)
Intercept	-1.67 (0.30)*	-1.93 (0.38)*	-3.48 (0.49)*	-1.77 (0.44)*
B-Lagoon:Present	0.26 (0.12)*	0.34 (0.2)	0.84 (0.28)*	-
B-Grass Height: Short	0.15 (0.12)	0.44 (0.15)*	-	-
B-Grass Height: Long	-0.05 (0.19)	-0.58 (0.61)	-	-
B-Grass Color: Green	-	0.76 (0.14)*	0.38 (0.21)	-
AB-Water: Presence	0.47 (0.14)*	-	-	1.29 (0.27)*
AB-Year:2013	-	-	0.34 (0.29)	0.14 (0.26)
AB-Year:2014	-	-	0.47 (0.29)	-0.26 (0.27)
AB-Year:2015	-	-	0.97 (0.32)*	-0.96 (0.34)*
AB-Season: Mid	0.05 (0.12)	-	0.02 (0.27)	-
AB-Season: Late	0.31(0.11)*	-	0.47 (0.23)*	-
AB-Burn: Presence	-0.05 (0.15)	-0.49 (0.29)	-	-
p - Covariates	β Estimate (SE)	β Estimate (SE)	β Estimate (SE)	β Estimate (SE)
Intercept	-3.14 (0.12)*	-2.26 (0.18)*	-2.78 (0.3)*	-3.03 (0.42)*
B-Closed Woodland	0.02 (0.17)	-	0.72 (0.35)*	-0.24 (0.35)
B-Open Scrubland	0.43 (0.11)*	-	0.21 (0.32)	0.2 (0.31)
B-Open Woodland	0.83 (0.11)*	-	1.1 (0.29)*	0.58 (0.3)
B-Open Grassland Flooded	0.98 (0.13)*	-	2.18 (0.93)*	1.27 (0.43)*
B-Open Grassland Not Flooded	1.06 (0.19)*	-	0.94 (0.5)	0.66 (0.36)
B-Woodland	-	0.67 (0.17)*	-	-
B-Grassland	-	0.55 (0.14)*	-	-
A-Seasonal Track	-	-0.25 (0.18)	-0.82 (0.33)*	-
A-Permanent Track	-	-0.33 (0.14)*	-0.46 (0.23)*	-
Hazard Detection Function	0.86 (0.08)*	0.85 (0.13)*	0.83 (0.23)*	0.74 (0.26)*

Like herd density models, herd size models and coefficient estimates varied between species (Table 5). Segments on the western side of the Luangwa River were associated with smaller herds for impala (21% smaller) and puku (16% smaller), while neither side of the Luangwa River nor protection status were detectably correlated with zebra or warthog herd sizes. Impala and puku herds were smallest in 2012, with detectably larger herds during the remainder of the study period (except 2014 for puku).

Table 5: Log-link coefficient estimates and standard errors from final herd size models. Change values are the multiplicative changes of group size based on the associated coefficient estimate, derived using the *predict()* function. Covariates not included in the final models are indicated as either dropped during model refinement (+) or excluded from model refinement due to high correlation or imbalanced sampling (-).

	Impala		Puku		Zebra		Warthog	
Covariate	Estimate (SE)	Change	Estimate (SE)	Change	Estimate (SE)	Change	Estimate (SE)	Change
Intercept	0.612 (0.288)*		-0.252 (0.570)		1.977* (0.107)		1.294 (0.103)*	
Mixed Species Herd	0.563 (0.091)*	1.72	0.617 (0.172)*	1.27	0.228* (0.081)	1.25	+	+
B-Edge Density	0.037 (0.005)*	1.03	+	+	-0.024* (0.004)	0.98	+	+
B-Lagoon:Present	+	+	1.588 (0.378)*	2.47	+	+	+	+
B-Log(% CS)	0.028 (0.009)*	1.02	+	+	+	+	+	+
B-% CW	-	-	-	-	-	-	-	-
B-% OW	0.004 (0.002)*	1.00	-	-	+	+	+	+
B-% OG	-	-	0.008 (0.003)*	1.00	-	-	+	+
B-Grass Height: Short	0.194 (0.081)*	1.19	+	+	0.360* (0.100)	1.42	+	+
B-Grass Height: Long	-1.949 (0.478)*	0.38	+	+	0.230 (0.132)	1.25	+	+
B-Grass Color: Green	-0.562 (0.103)*	0.58	-0.357 (0.173)*	0.88	+	-	-0.469 (0.117)*	0.71
T-Lion UD	-	-	-	-	+	+	-	-
A-Log Distance to Roads	+	+	+	+	+	+	+	+
A-Side: West	-0.275 (0.100)*	0.79	-0.807 (0.180)*	0.84	-	-	+	+
A-Area: Park	-	-	-	-	+	+	-	-
AB-Distance to Luangwa	0.126 (0.020)*	1.12	-0.169 (0.087)	0.96	-	-	-	-
AB-Distance to Seasonal Stream	0.067 (0.017)*	1.06	-	-	+	+	-0.097 (0.029)*	0.942
AB-Water: Presence	0.786 (0.100)*	1.77	-	-	+	+	+	+
AB-Year:2013	0.517 (0.125)*	1.64	0.844 (0.290)*	1.43	+	+	+	+
AB-Year:2014	0.339 (0.111)*	1.38	0.293 (0.305)	1.10	+	+	+	+
AB-Year:2015	0.268 (0.132)*	1.29	0.661 (0.324)*	1.30	+	+	+	+
AB-Season: Mid	0.319 (0.104)*	1.35	0.244 (0.184)	1.08	-0.209 (0.107)	0.82	+	+
AB-Season: Late	-0.086 (0.103)	0.93	-0.401 (0.248)	0.91	-0.210* (0.086)	0.81	+	+
AB-Burn: Presence	-	-	-	-	0.303* (0.087)	1.35	+	+

3.2 Population Density

Impala had the highest population density, with an average density of 27.69 animals/km^2, and showed great spatiotemporal variation in density (range among segments: 0.24 – 601.18 animals/km^2). Puku also

occurred at variable densities, an average density of 8.11 animals/km^2 (range: 0.003 – 172.60 animals/km^2). Zebra and warthog occurred at lower and less variable densities, with average densities of 2.41 animals/km^2 (range: 0.03– 28.09 animals/km^2) and 1.76 animals/km^2 (range: 0.05– 13.82 animals/km^2), respectively.

3.2.1 Protection effects

Herbivore densities varied widely across the SLPG, and the highest densities for all species were within the fully-protected SLNP (Figure 2). Within SLNP, density varied between portions of the park that were west and east of Luangwa River. Impala and zebra densities did not detectably differ within these two areas of the national park, while both puku and warthog occurred at their highest densities in eastern parklands. Thus, there were no consistent differences between areas east and west of the river that had the same legal protection. All species occurred at lower densities in the GMA, ranging from 4.5-fold to 13.2-fold lower than densities within SLNP, with no overlap in 95% CIs.

Fig 2: Population density estimates across the South Luangwa protection gradient. Regions are arranged left to right in level of protection, with west and east denoting side of the Luangwa River. Points indicate segment-specific density estimates across the four-year study, overlaid with average annual densities and 95% CIs. Overall regional average densities (diamonds) and 95% CIs are displayed in bold alongside the annual averages. Y-axes were truncated to clearly display variation between annual averages, and thus omit extreme segment estimates from being displayed (13 impala, 16 puku, 4 Zebra, and 3 warthog estimates out of 890 total estimates for each species).

3.2.2 Temporal effects

Density estimates varied among years, but annual differences from regional mean densities were species-specific, with no consistent pattern for across species (Figure 2). Impala density did not detectably change across the study period in any section of the protection gradient. Puku density increased from 2012 to 2013 in western parklands but did not differ from 2014 and 2015 estimates or the overall mean density for

that region. Zebra densities did not change in GMA and eastern parklands but increased in western parklands. Finally, we detected a decline in warthog densities across all regions in the protection gradient.

3.2.3 Predicted population density across the SLPG and the study period

After controlling for all covariates, we found that the observed differences in population density just described were not always detectably related to the level of protection (Figure 3) or year (Figure 4). Across the SLPG, predicted mean species densities were consistently higher in protected areas, but some of these estimated differences had low precision (Figure 3). For impala, populations in protected parklands east and west of the Luangwa River were estimated to have 1.3- and 0.66-fold greater densities compared to GMA densities, but all CIs showed that predicted mean density overlapped with GMA estimates. Puku densities were 9.5-fold greater in the eastern parklands (with >80% confidence that a difference existed due to protection), while densities in western parklands were not detectably different from the GMA. Predicted zebra densities were 5-fold and 12-fold greater in the eastern parklands and western parklands, respectively, with high confidence that a protection effect existed for both comparisons. Predicted warthog densities were higher in protected regions compared to the GMA, but CIs overlapped predicted GMA densities. Throughout the study period we could not detect any density trends for any species after controlling for other covariates (Figure 4), including during the trophy hunting moratorium (2013-2014).

Fig 3: Differences in population density estimates between protected park lands and GMA population density estimates (dotted line), with other covariates held constant. Error bars indicate 80%, 90%, and 95% confidence limits.

Fig 4: Differences in population density estimates through the study period and 2012 density estimates (dotted line), with other covariates held constant. Error bars indicate 80%, 90%, and 95% confidence limits.

In summary: (1) Ungulate densities were consistently higher in the better protected SLNP. (2) There was evidence that higher protection status yields higher density, after controlling for ecological and abiotic differences between NP and GMA, but this evidence was mixed and sometimes not strong. (3) After controlling for other effects, there was no consistent pattern of change in ungulate densities over the four years of observation.

4. Discussion

Our study of the SLPG supports previous findings that lands with better protection generally hold higher densities of herbivores [14-17], supporting continued protection and monitoring in the SLPG. In particular, the segment of SLNP east of the Luangwa River still supports densities of ungulates comparable to or greater than in SLNP west of the Luangwa River, despite having no physical boundary to prevent human incursions and a heavily-used public road passing through it. We estimated the lowest densities for all focal species in the SLPG within the GMA, which supports conclusions from previous studies in the SLPG that suggested depleted herbivore populations within Lupande GMA [23,26]. Given that our GMA transects surveyed areas within 6 km of the Luangwa River, where wildlife densities are thought to be highest and away from dense human settlements, the densities we estimated within this buffer area are likely high compared to the rest of the GMA. These inferences are restricted to dry season conditions, as we were not able to survey transects in wet season conditions.

However, after controlling for a suite of bottom-up, top-down, anthropogenic, and abiotic covariates, there is no clear evidence that these dynamics were driven by protection status or year alone. The non-anthropogenic variables included in our analysis incorporate ecosystem alteration by humans, and thus our interpretation of the role of the SLPG regions and time are proxies for anthropogenic mortality and associated risk effects [48]. Isolating these forces from ecological variables is not entirely clear; for example, poaching efforts are non-random and are correlated with both anthropogenic and ecological variables [27]. We did not allow trends to vary across the SLPG, so any differences in trend between regions for a species are due to covariates other than year (Figure 2), supported by lacking trends when all

covariates other than year were held constant. With these considerations, our findings indicate that an array of ecological and anthropogenic variables influence herbivores in protected area networks characterized by national parks (or other strictly protected areas) and adjoining buffer zones.

If we had not applied our rigorous modeling of both group density and size, and instead followed previous approaches (see Section 1), our inferences would have been confined to the variables of interest (protection and year) and would not acknowledge the effects of other important variables. With our approach, ecological variables could play an important role in our modeling process, highlighting the importance of considering ecological conditions in the SLPG along with the protections provided by SLNP. Herbivore density can vary across gradients of protection, but our findings indicate that the protection status of an area is generally insufficient to capture the complexities of factors influencing herbivores across this protection gradient.

4.1 Differences in Density across the South Luangwa Protection Gradient

Illegal wire snare poaching has been a well-recognized threat to wildlife communities in the Luangwa Valley [49] and has been identified as a major threat to the persistence of large carnivores [26]. In this study, GMA transects represented areas that were prone to high risk for snare occurrence relative to risk in SLNP due to proximity to human activity and greater law enforcement efforts within SLNP [27]. Therefore, we would expect reduced herbivore densities in GMA relative to SLNP regions if bushmeat poaching was impacting herbivore densities. After controlling for all other variables, our density estimates were higher in PA regions of the SLPG, but only zebra densities reflected this expectation with 95% confidence (puku reflected this expectation with 80% confidence; Figure 3). The detected role of the SLPG in zebra density dynamics indicates evidence of increased impacts of bushmeat poaching, particularly as larger ungulate species are vulnerable to overharvest [3]. Other sources of mortality are unlikely drivers of zebra density dynamics in this case. Trophy hunting occurred with limited quotas and small harvest rates relative to our population density estimates, and lion utilization was accounted for in our modeling of group density and size (Table 4). Therefore, the predictable impact of wire-snare

poaching on zebra (and likely puku) is a signal for concern but demonstrates that the protection gradient is providing protection from human exploitation.

Despite concerns of increased poaching in the absence of wildlife-based economies during the trophy hunting moratorium in GMAs, there is no evidence of any coinciding large-scale herbivore decline in our study area. Anti-poaching efforts in the study area were conducted jointly by the Department of National Parks and Wildlife and Conservation South Luangwa during this period, and the level of anti-poaching effort and support increased during the moratorium [29,50-52]. We acknowledge that this is not necessarily the case in all concessions but does indicate the potential for greater anti-poaching investment by operators in these areas. We did not test for interactions between year and region of the protection gradient, so our estimates of population trends could hide local declines in the GMA. However, these results indicate no evidence that the 2013-2014 trophy moratorium was detrimental to the species in this study.

Estimated densities across the SLPG and throughout the study period are clearly influenced by ecological variables, indicating that habitat alteration likely plays an important role in decreasing available resources and reducing herbivore densities in the GMA. Colleagues [19] documented rapid rates of human encroachment in Lupande and other GMAs around key Zambian protected areas. Our GMA transects did not pass directly through any majorly altered areas (e,g, agriculture or villages), but such areas were present across the GMA region of the SLPG. In addition to human encroachment, altered composition of the wildlife community in the GMA, particularly the poaching of elephants and rhinos (extirpated by 1995) [53], likely influences vegetation structure in this region [54]. The role of habitat conversion and changing wildlife community composition should be further investigated in the SLPG, alongside ongoing attention to combat illegal bushmeat poaching.

4.2 The future role of ground transects in the South Luangwa Protection Gradient

Future monitoring is critical to track population densities for these and other herbivore species, particularly in the face of a growing human population. While multiple approaches are implemented to achieve this monitoring, we advocate continued monitoring by stratified, ground-based transects. Aerial survey data collected by the Zambia Department of National Parks and Wildlife supports the notion that the SLPG supports notably high densities for all four species [55], and therefore remains an important area to protect and monitor. While ground-based distance sampling surveys cannot match the spatial scale achievable by aircraft, ground-based surveys provide more accurate density estimates to identify population trends and aid in our understanding of the function of protected areas. The difference in costs between the two methods is well-documented [56], and population monitoring could be supplemented with data collected by law enforcement patrols [57] and citizen science initiatives [58] to offset costs and to achieve a large study area. Ground-based distance sampling methods should be integrated into long-term monitoring of the SLPG and in other protected areas, focused on critical areas for ungulate populations and accounting for the dynamics of ecological covariates in both group density and size, as demonstrated in this study.

Acknowledgements

We thank the Zambia Department of National Parks and Wildlife for permission and collaboration with this study. Many thanks to G. Banda, T. Banda, B. Beza, R. Kabungo, A. Mwanza, A. Rosenblatt, E. Sadowski, and M. Sichande for their assistance with data collection.

References

1. Gordon IJ, Hester AJ, Festa- Bianchet M. Review: the management of wild large herbivores to meet economic, conservation and environmental objectives. J Appl Ecol 2004;41: 1021-1031.

2. Western D, Russell S, Cuthill I. The status of wildlife in protected areas compared to non-protected areas of Kenya. PloS one 2009; 4:e6140.

3. Ripple WJ, Newsome TM, Wolf C, Dirzo R, Everatt KT, Galetti M, et al. Collapse of the world's largest herbivores. Sci Adv 2015;1:e1400103.

4. Wittemyer G, Elsen P, Bean WT, Burton ACO, Brashares JS. Accelerated human growth at protected area edges. Science 2008;321: 123-126.

5. Geldmann J, Barnes M, Coad L, Craigie ID, Hockings M, Burgess ND. Effectiveness of terrestrial protected areas in reducing habitat loss and population declines. Biol Conserv 2013;161: 230-238.

6. Woodroffe R, Ginsberg J. Edge effects and the extinction of populations inside protected areas. Science 1998;280: 2126-2128.

7. Pulliam HR. Sources, sinks, and population regulation. Am Nat 1988;132: 652–661.

8. Joppa L, Pfaff A. Reassessing the forest impacts of protection: the challenge of nonrandom location and a corrective method. Ann. N.Y. Acad. Sci. 2010;1185: 135–149.

9. M'soka J, Creel S, Becker MS, Murdoch JD. Ecological and anthropogenic effects on the density of migratory and resident ungulates in a human-inhabited protected area. Afr J Ecol 2017;55: 618–631.

10. Buckland ST. Introduction to Distance Sampling: Estimating Abundance of Biological Populations. Oxford: Oxford University Press; 2001.

11. Royle JA, Dawson DK, Bates S. Modeling abundance effects in distance sampling. Ecol 2004;85: 1591-1597.

12. Ferguson MC, Barlow J, Fiedler P, Reilly SB, Gerrodette T. Spatial models of delphinid (family Delphinidae) encounter rate and group size in the eastern tropical Pacific Ocean. Ecol Model 2006;193: 645–662.

13. Rduch V. Population characteristics and coexistence of puku (Kobus vardonii) and impala (Aepyceros melampus) in and around Kafue National Park, Zambia. Mamm Biol 2016;81: 350-360.

14. Caro TM, Pelkey N, Borner M, Campbell KLI, Woodworth BL, Farm BP, et al. Consequences of different forms of conservation for large mammals in Tanzania: preliminary analyses. Afr J Ecol 1998;36: 303–320.

15. Stoner C, Caro T, Mduma S, Mlingwa C, Sabuni G, Borner M. Assessment of effectiveness of protection strategies in Tanzania based on a decade of survey data for large herbivores. Conserv Biol 2007;21: 635–646.

16. Ogutu JO, Piepho HP, Dublin HT, Bhola N, Reid RS. Dynamics of Mara–Serengeti ungulates in relation to land use changes. J Zool 2009;278: 1–14.

17. Schuette P, Creel S, Christianson D. Ungulate distributions in a rangeland with competitors, predators and pastoralists. J Appl Ecol 2016;53: 1066-1077.

18. Purchase G, Mateke C, Purchase D. A review of the status and distribution of carnivores, and levels of human- carnivore conflict, in the protected areas and surrounds of the Zambezi Basin. Unpublished report. The Zambezi Society, Bulawayo. 2007. pp 1-77.

19. Watson FGR, Becker MS, Milanzi J, Nyirenda M. Human encroachment into protected area networks in Zambia: implications for large carnivore conservation. Reg Environ Change 2014;15: 415-429.

20. Rosenblatt E, Becker MS, Creel S, Droge E, Mweetwa T, Schuette PA et al. Detecting declines of apex carnivores and evaluating their causes: An example with Zambian lions. Biol Conserv 2014;180: 176–186.

21. Lindsey PA, Nyirenda VR, Barnes JI, Becker MS, Mcrobb R, Tambling CJ, et al. Underperformance of African protected area networks and the case for new conservation models : Insights from Zambia. PLoS One 2014;9: e94109

22. Becker MS, Watson FGR, Droge E, Leigh K, Carlson RS, Carlson AA. Estimating past and future male loss in three Zambian lion populations. J Wildl Manag 2013;77: 128–142.

23. Rosenblatt E, Creel S, Becker MS, Merkle J, Mwape H, Schuette P, Simpamba T. Effects of a protection gradient on carnivore density and survival: an example with leopards in the Luangwa valley, Zambia. Ecol Evol 2016;6: 3772-3785.

24. Creel S, M'soka J, Dröge E, Rosenblatt E, Becker M, Matandiko W, Simpamba T. Assessing the sustainability of African lion trophy hunting, with recommendations for policy. Ecol Appl 2016;26: 2347-2357.

25. Mweetwa T, Christianson D, Becker MS, Creel S, Rosenblatt E, Merkle J, et al. Quantifying lion demographic responses during a three-year moratorium on trophy hunting. PLOS One 2018;13: e0197030.

26. Becker MS, McRobb R, Watson F, Droge E, Kanyembo B, Murdoch J, Kakumbi C. Evaluating wire-snare poaching trends and the impacts of by-catch on elephants and large carnivores. Biol Conserv 2013;158: 26–36.

27. Watson F, Becker MS, McRobb R, Kanyembo B. Spatial patterns of wire-snare poaching: implications for community conservation in buffer zones around National Parks. Biol Conserv 2013;168: 1–9.

28. White PA, Belant JL. Provisioning of Game Meat to Rural Communities as a Benefit of Sport Hunting in Zambia. PloS one 2015;10: e0117237.

29. SLCS. South Luangwa Conservation Society Annual Report 2014. 2015. 22 pp.

30. Astle WL, Webster R, Lawrance CJ. Land classification for management planning in the Luangwa Valley of Zambia. J Appl Ecol 1969;6: 143–169.

31. White F. The Vegetation of Africa: A Descriptive Memoir to Accompany the Unesco/AETFAT/UNSO Vegetation Map of Africa. Paris, France: United Nations Educational, Scientific and Cultural Organization; 1983.

32. Republic of Zambia. 2010 census of population and housing. Lusaka, Zambia; Central Statistical Office; 2011.

33. ZAWA. Conservation Action Plan for the Lion in Zambia. Chilanga, Zambia:Zambia Wildlife Authority; 2010.

34. Maechler M, Rousseeuw P, Struyf A, Hubert M, Hornik K. cluster: Cluster Analysis Basics and Extensions. R package version 2.0.4; 2016.

35. R Core Team. R: A language and environment for statistical computing. Vienna, Austria: R Foundation for Statistical Computing; 2019.

36. Pebesma EJ, Bivand RS. Classes and methods for spatial data in R. R News 5; 2005.

37. Bivand RS, Pebesma E, Gomez-Rubio V. Applied spatial data analysis with R. 2nd ed. New York: Springer; 2013.

38. Bivand RS, Keitt T, Rowlingson B. rgdal: Bindings for the Geospatial Data Abstraction Library. R package version 1.0-4; 2015.

39. Wickham H. The Split-Apply-Combine Strategy for Data Analysis. J Stat Softw 2011;40: 1-29.

40. Dröge E, Creel S, Becker M, M'soka J. Spatial and temporal avoidance of risk within a large carnivore guild. Ecol Evol 2017;7: 189-199.

41. Calenge C. The package adehabitat for the R software: a tool for the analysis of space and habitat use by animals. Ecol Model 2006;197: 516-519.

42. Hijmans RJ. raster: Geographic Data Analysis and Modeling. R package version 2.4-15; (2015).

43. Anderson DR, Laake JL, Crain BR, Burnham KP. Guidelines for line transect sampling of biological populations. J Wildl Manag 1979;43: 70-78.

44. Wilsey BJ. Variation in use of green flushes following burns among African ungulate species: the importance of body size. Afr J Ecol 1996;34: 32-38.

45. Chandler RB, Royle JA, King DI. Inference about density and temporary emigration in unmarked populations. Ecol 2011;92: 1429–1435.

46. Zuur AF, Leno EN, Walker NJ, Savaliev AA, Smith GM. Mixed effects models and extensions in ecology with R. New York: Springer; 2009.

47. Yee TW. Vector Generalized Linear and Additive Models: With an Implementation in R. New York: Springer; 2015.

48. Frid A, Dill L. Human-caused disturbance stimuli as a form of predation risk. Conserv Ecol 2002;6: 11.

49. Lewis DM, Phiri A. Wildlife snaring—an indicator of community response to a community-based conservation project. Oryx 1998;32: 111–121.

50. SLCS. Annual Report to Luangwa Conservation Community Fund. 2012. 22 pp.

51. SLCS. South Luangwa Conservation Society Annual Report 2013. 2014. 24 pp.

52. CSL. Conservation South Luangwa Annual Report 2015. 2015. 21 pp.

53. Nyirenda VR, Lindsey PA, Phiri E, Stevenson I, Chomba C, Namukonde N, et al. Trends in Illegal Killing of African Elephants (Loxodonta africana) in the Luangwa and Zambezi Ecosystems of Zambia. Environ Nat Resour Res 2015;5: 24-36.

54. Sankaran M, Augustine DJ, Ratnam J. Native ungulates of diverse body sizes collectively regulate long- term woody plant demography and structure of a semi- arid savanna. J Ecol 2013;101: 1389-1399.

55. DNPW. Report on the 2015 aerial census of elephants and other large mammals in Zambia: Volume II Population estimates for other large mammals and birds. Chilanga, Zambia: Department of National Parks and Wildlife; 2016.

56. Jachmann H. Estimating Abundance of African Wildlife: An Aid to Adaptive Management. Boston: Kluwer Academic Publishers; 2001.

57. Schuette P, Namukonde N, Becker MS, Watson F, Creel S, Chifunte C, et al. Boots on the ground: in defense of low‐ tech, inexpensive, and robust survey methods for Africa's under‐ funded protected areas. Biodivers Conserv 2018;27: 2173-2191.

58. Marnewick K. Conservation biology of cheetahs Acinonyx jubatus (Schreber 1775) and African wild dogs Lycaon pictus (Temminck 1820) in South Africa. Doctoral Dissertation, University of Pretoria, South Africa. 2016.

Figure 1

Click here to download Figure Fig1.tif

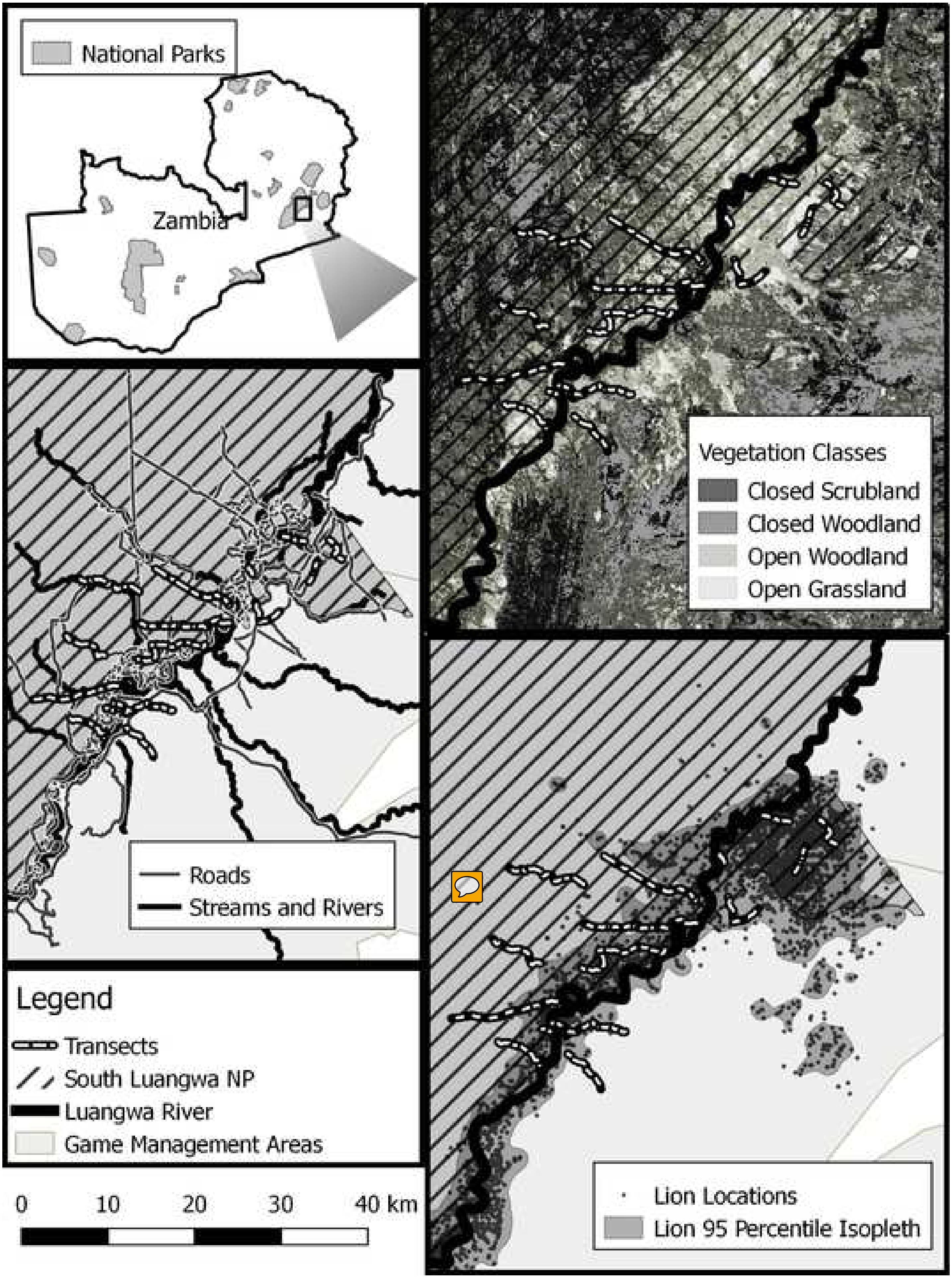

Figure 2

Click here to download Figure Fig2.tif

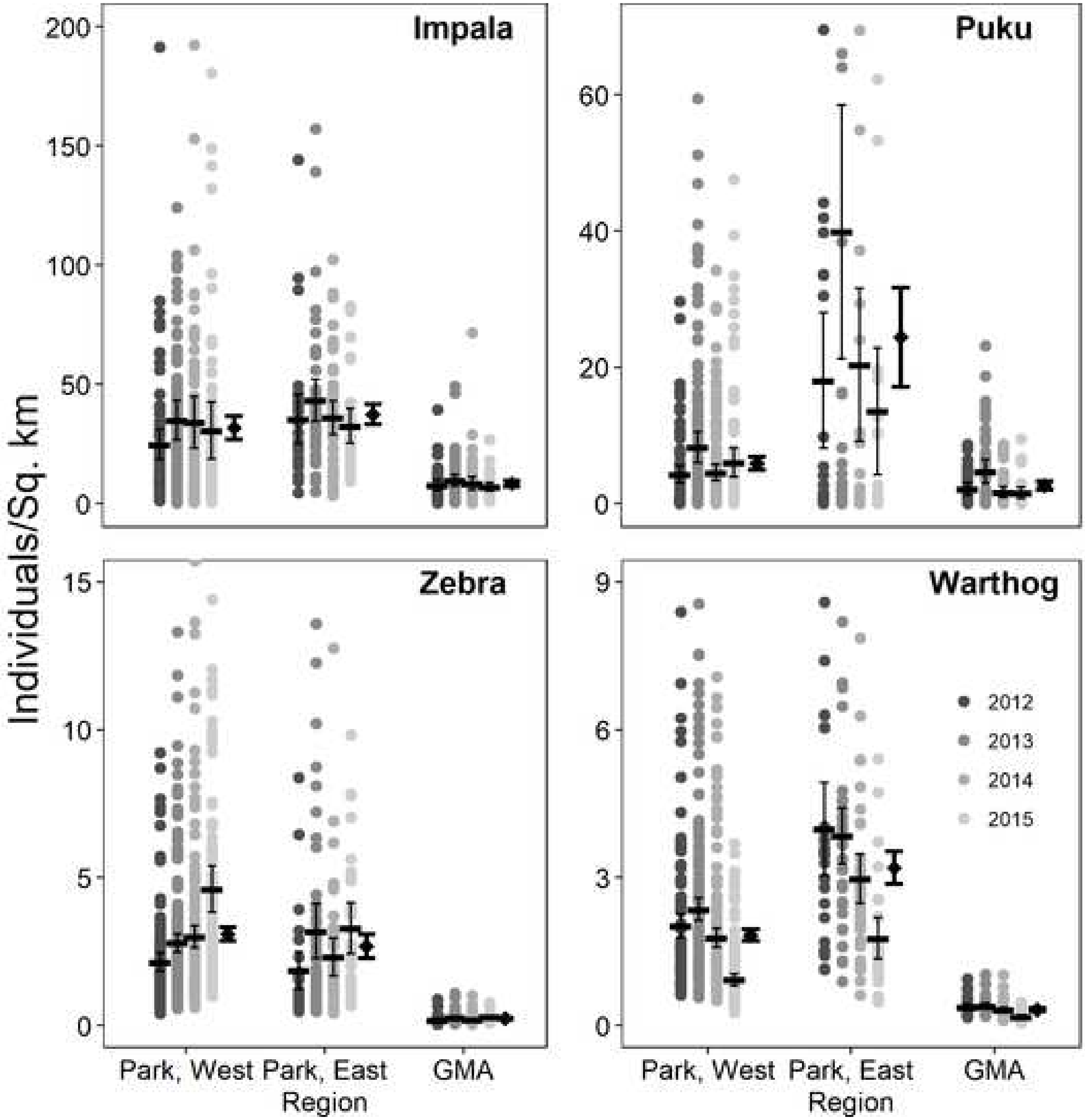

Figure 3

Click here to download Figure Fig3.tif

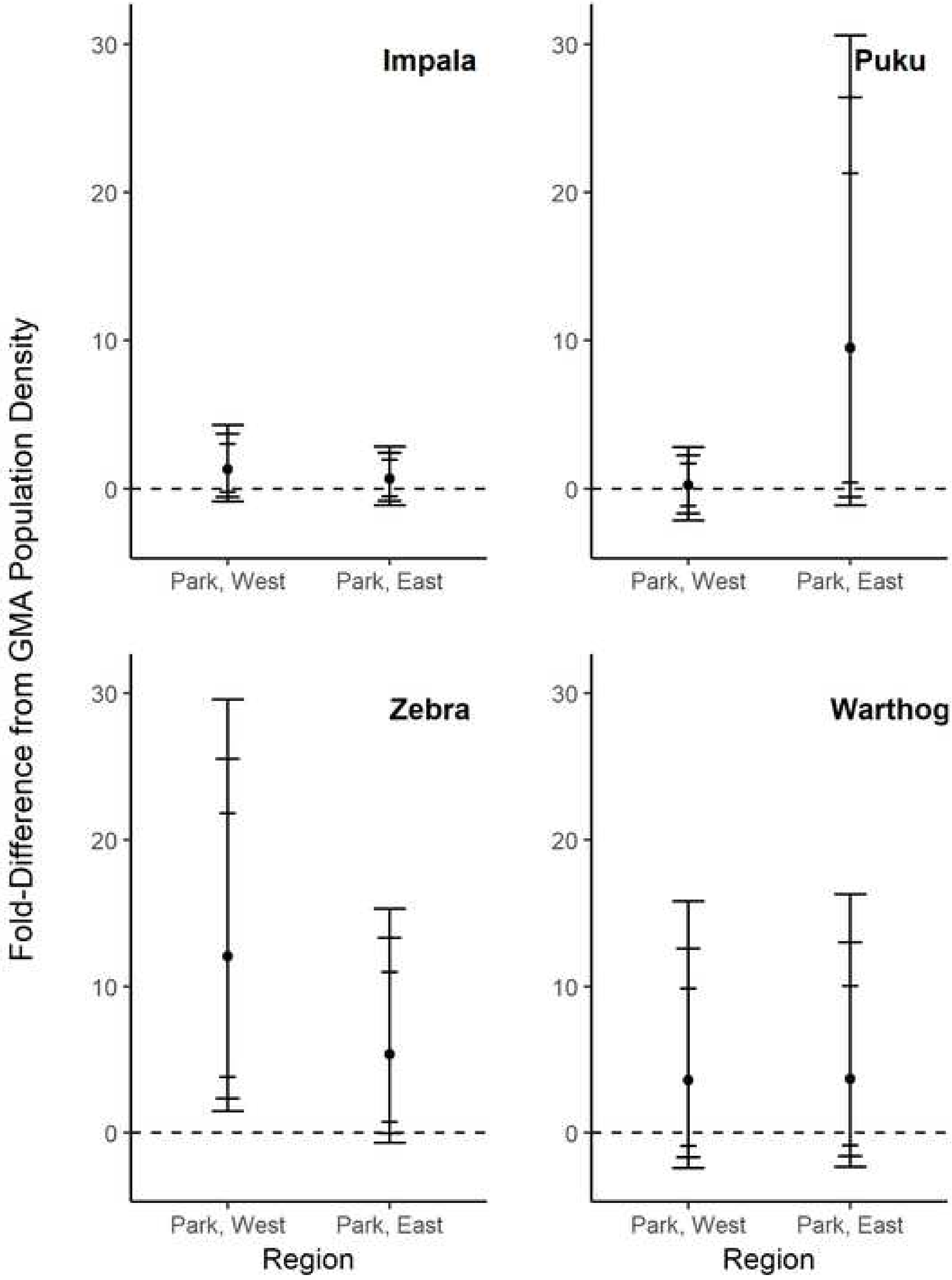

Figure 4

Click here to download Figure Fig4.tif

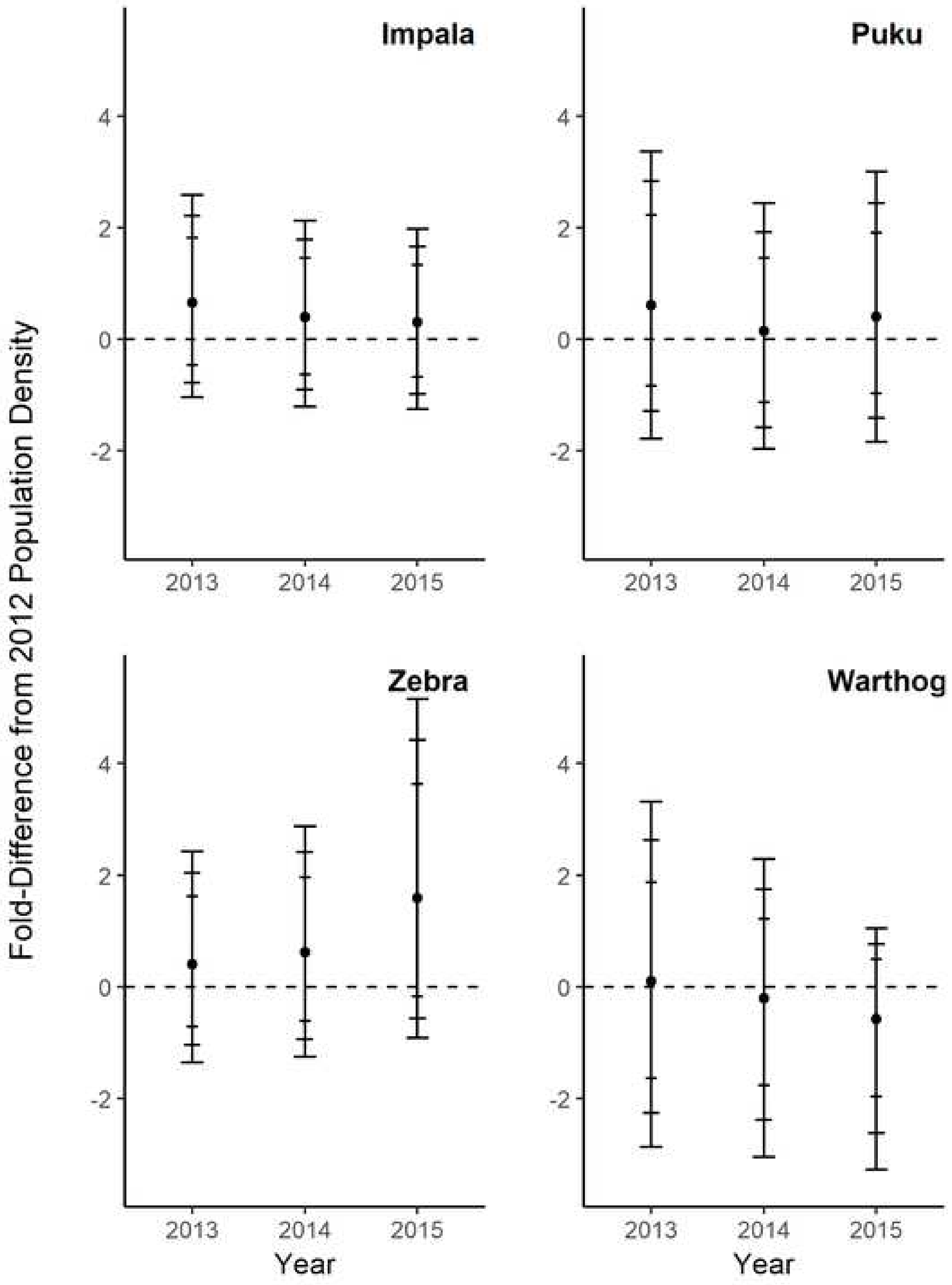

Supplement: Attachment — Submitted filename: PONE-D-19-19082_reviewer (1)-1.pdf [file pone.0224438.s005.pdf]
