# Supplementary material for: Do protection gradients explain patterns in herbivore densities? An example with ungulates in Zambia’s Luangwa Valley
Source: PLoS One. 2019 Oct 30;14(10):e0224438. doi: 10.1371/journal.pone.0224438 (PMC6821096; doi:10.1371/journal.pone.0224438)
Supplement: Attachment — Submitted filename: Rosenblatt Plos One review.docx [file pone.0224438.s003.docx]

I reviewed ‘Do protection gradients explain patterns in herbivore densities? An example with ungulates in Zambia’s Luangwa Valley’ which was submitted to Plos One by Rosenblatt et al. The paper examines temporal and spatial changes in population estimates of 4 species in Africa to determine whether protection (i.e., a ban on hunting) affects animal density. Overall, the paper was very well written and was very statistically thorough. There are a few items that warrant further explanation/clarification.

1. Authors conducted transect based distance sampling via vehicles in 3 regions – the National Park, a subset of the National Park where poaching was thought to be occurring, and a Game Management Area. The animals in the National Park will be accustomed to vehicular traffic due to the frequent photography safaris whereas the animals in the Game Management Area will likely associate vehicles with hunting activities. How would the different behavior among sites influence analysis, particularly the density estimates?
2. Given that density may be a misleading indicator of habitat quality (Van Horne 1983), I am surprised authors did not supplement data with recruitment data. Data such as adult female:offspring ratios would be easy to obtain during surveys and would elucidate factors contributing towards different density estimates.
3. Would it be possible for distribution of the 4 animals to be widespread during the wet season and become concentrated near the river during the dry season when your surveys took place? If animals do become concentrated, would it be possible for the animals that were East of the National Park to cross the river and enter the National Park where human disturbance is presumably lower and potentially inflate density estimates in the National Park? Some supporting information that describes site fidelity (or lack thereof) of the 4 focal species would be helpful.
4. A few more minor comments can be found within the pdf.
